# Expression of centromere protein F (CENP-F) associated with higher FDG uptake on PET/CT, detected by cDNA microarray, predicts high-risk patients with primary breast cancer

**DOI:** 10.1186/1471-2407-8-384

**Published:** 2008-12-22

**Authors:** Shigeto Ueda, Nobuo Kondoh, Hitoshi Tsuda, Souhei Yamamoto, Hideki Asakawa, Kazuhiko Fukatsu, Takayuki Kobayashi, Junji Yamamoto, Katsumi Tamura, Jiro Ishida, Yoshiyuki Abe, Mikio Yamamoto, Hidetaka Mochizuki

**Affiliations:** 1 Department of Surgery, National Defense Medical College, 3-2 Namiki, Tokorozawa, Saitama 359-8513, Japan; 2 Department of Oral Biochemistry, Asahi University School of Dentistry, 1851 Hozumi, Mizuho, Gifu, 501-0296, Japan; 3 Department of Basic Pathology, National Defense Medical College, 3-2 Namiki, Tokorozawa, Saitama 359-8513, Japan; 4 Tokorozawa PET Diagnostic Imaging Clinic, 7-5 Higashi-Sumiyoshi, Tokorozawa, Saitama 359-1124, Japan; 5 Department of Biochemistry, National Defense Medical College, 3-2 Namiki, Tokorozawa, Saitama 359-8513, Japan

## Abstract

**Background:**

Higher standardized uptake value (SUV) detected by 18F-fluorodeoxyglucose positron emission tomography/computed tomography (FDG PET/CT) correlates with proliferation of primary breast cancer. The purpose of this study is to identify specific molecules upregulated in primary breast cancers with a high SUV and to examine their clinical significance.

**Methods:**

We compared mRNA expression profiles between 14 tumors with low SUVs and 24 tumors with high SUVs by cDNA microarray. We identified centromere protein F (CENP-F) and CDC6 were upregulated in tumors with high SUVs. RT-PCR and immunohistochemical analyses were performed to validate these data. Clinical implication of CENP-F and CDC6 was examined for 253 archival breast cancers by the tissue microarray.

**Results:**

The relative ratios of CENP-F and CDC6 expression levels to β-actin were confirmed to be significantly higher in high SUV tumors than in low SUV tumors (*p *= 0.027 and 0.025, respectively) by RT-PCR. In immunohistochemical analysis of 47 node-negative tumors, the CENP-F expression was significantly higher in the high SUV tumors (74%) than the low SUV tumors (45%) (*p *= 0.04), but membranous and cytoplasmic CDC6 expressions did not significantly differ between both groups (*p *= 0.9 each). By the tissue microarray, CENP-F (HR = 2.94) as well as tumor size (HR = 4.49), nodal positivity (HR = 4.1), and Ki67 (HR = 2.05) showed independent impact on the patients' prognosis.

**Conclusion:**

High CENP-F expression, correlated with high SUV, was the prognostic indicators of primary breast cancer. Tumoral SUV levels may serve as a pretherapeutic indicator of aggressiveness of breast cancer.

## Background

Although a majority of patients with primary breast cancer are cured by locoregional treatment with or without systemic therapy, approximately 10–30% of these patients experience recurrence of the cancer [1]. Researchers have suggested various biomarkers or risk categories of breast cancer to reliably identify high-risk patients, who would require chemotherapy, and low-risk patients, who can be treated without chemotherapy [2]. Nodal status is the important prognostic and predictive factors in the treatment of primary breast cancer; however, there are still no definitive criteria that can identify high-risk patients with primary breast cancer, especially those with node-negative (pN0) breast cancer.

Currently, primary systemic therapy (PST) is widely practiced as a standard therapy for patients with early-stage breast cancer [3], but it is difficult to preoperatively identify high-risk patients in the PST setting because available information obtained from core needle biopsy specimens is limited. Although molecular biology tools such as Oncotype DX™ or the 70-gene expression classifier identified by DNA microarray analysis (MammaPrint^®^) are going to be used widely for risk evaluation, utility of these tools has not been confirmed with primary breast cancer in the PST setting [4-6]. Therefore, we need new tools that can preoperatively and accurately predict the prognosis of patients with early-stage breast cancer.

FDG PET/CT is a noninvasive imaging device widely used for the detection and staging of breast cancer[7]. FDG uptake on PET, quantified by the standardized uptake value (SUV), is a highly reproducible parameter of tumor glucose metabolism [8].

We previously reported that high SUV levels (4.0 or more) in primary breast tumors significantly correlated with higher nuclear grades and poorer prognoses of primary breast tumors [9]. We suggested that the tumoral SUV detected by FDG PET/CT could be a preoperative tool to predict high-risk patients with primary breast cancer.

In fact, primary cancers with high SUV detected by FDG PET/CT correlated with the parameters of rapid cellular proliferation, e.g., mitotic counts and Ki67 index, and exhibited poorer clinical outcome than those with low SUV. Such correlation was reported in various cancers, including non-small-cell lung cancer, ovarian cancer, and breast cancer [10-13]. When novel prognostic molecules correlated with high SUV are identified, it might be very helpful for the accurate preoperative prediction of high-risk patients with early breast cancer.

The present study aimed at detecting novel candidate genes from primary breast cancers with high SUV. We compared mRNA expression profiles and protein expressions of several specific molecules between primary breast cancer with high SUV and those with low SUV by means of cDNA microarray and immunohistochemistry. We also evaluated whether the candidate genes identified by the cDNA microarray correlated with the clinical outcome. Furthermore, we discussed whether the SUV categories by FDG PET/CT are useful to predict tumor proliferation and to identify patients having high-risk primary breast cancer before the systemic therapy or surgery.

## Methods

### Patient characteristics

This study was approved by the institutional review committee of the National Defense Medical College, Japan. Informed consent was obtained from each eligible patient. Primary breast cancer was histopathologically diagnosed on a core needle biopsy. Patients who did not exhibit evidence of distant metastatic spread in results of X-ray, ultrasonography, or FDG PET/CT were eligible as operable candidates for the treatment of primary breast cancer. Pregnant patients, patients with a history of insulin-dependent diabetes mellitus from clinical notes, or those who had previously received treatment to breast cancer were excluded from assessment with FDG PET/CT. These patients did not receive any systemic therapy before surgery. FDG PET/CT examination was performed at an interval of 2 weeks or more after the core needle biopsy (CNB). All the patients underwent surgery within 6 weeks after the FDG PET/CT examination.

Surgical specimens were obtained from patients who underwent mastectomy or lumpectomy for primary breast cancer. Initially, a total of 48 samples comprising 24 high SUV tumors and other 24 low SUV tumors, that were matched with regard to pT and pN factors, were subjected to RNA isolation. A sufficient volume of total RNA were extracted from all 24 samples of high SUV tumors and 14 (58%) of 24 samples of low SUV tumors. Therefore, a total of 38 samples were used for the cDNA microarray study.

These tissue samples of primary breast cancer in the 38 patients were subjected to cDNA microarray and quantitative RT-PCR analyses. The age of patients included in this study ranged from 36 to 81 years (mean, 56 years). The disease was in stage I in 18 patients (T1N0M0 in 18) and stage II in 20 (T1N1M0 in 4, T2N0M0 in 8, and T2N1M0 in 8). All the 38 tumors were histologically invasive ductal carcinoma. All patients underwent FDG PET/CT to evaluate the primary tumor and metastatic site of the disease.

### FDG PET/CT and quantification of FDG uptake in primary breast cancer

All patients underwent FDG PET/CT scans (Biograph LSO Emotion, Siemens) at the Tokorozawa PET Diagnostic Imaging Clinic (Tokorozawa, Japan). Patients fasted for at least 6 h before the FDG PET/CT study. One hour after the intravenous administration of 3.7 Mbq/kg FDG, a transmission scan using CT for attenuation correction and anatomical imaging was acquired for 90 s. Intravenous contrast was not administered to patients for the CT portion of the FDG PET/CT.

Back projection images were obtained after applying a Gaussian filter. The spatial resolution of the reconstructed images was 6.0–7.0 mm in the craniocaudal direction, 6.3–7.1 mm in the right-left direction, and 6.3–7.1 mm in the anterior-posterior direction.

SUV is defined as the decay-corrected tissue activity divided by the injected dose per patient body[9]. A region of interest (ROI) was placed in the primary lesion including the highest uptake area (circular ROI, 1 cm in diameter), and the SUV maximum in the ROI was calculated. SUV was calculated using the following formula: SUV = activity in ROI (MBq/ml)/injected dose (MBq/kg body weight).

We divided these 38 tumors into 2 groups based on the characteristics revealed in the FDG PET/CT. The low SUV group consisted of patients with a primary tumor with an SUV less than 4.0, whereas the high SUV group consisted of patients with a primary tumor with an SUV of 4.0 or greater. There were 14 tumors in the former group and 24 tumors in latter group.

Clinicopathological features of patients classified according to SUV are shown in Table 1. Regarding to age, pT-factor, pN-factor, nuclear grade, ER, and HER2, no statistical difference were detected between 2 groups.

**Table 1:** Clinicopathological features of 38 patients with primary breast cancer subjected to cDNA microarray and RT-PCR analyses

Number of patients				
Parameters		High SUV group (n = 24)	Low SUV group (n = 14)	*p*-value

SUV	Mean± SD	7.2± 2.5	2.3± 0.9	<0.0001
Age	Mean± SD	56.9± 14.1	59.8± 10.3	0.5
T-factor	T1	13	9	0.5
	T2	11	5	
N-factor	N0	16	10	0.8
	N+	8	4	
Nuclear grade	1,2	10	11	0.06
	3	14	3	
ER	-	7	1	0.1
	+	17	13	
HER2	0,1+,2+ (FISH-)	19	13	0.3
	3+ or FISH+	5	1	

Abbreviations; SUV, Standardized uptake value; ER, Estrogen receptor; HER2, c-erbB2; FISH, Fluorescence in situ hybridization; SD, standard deviation

### RNA extraction and cDNA microarray analysis

The tissue samples obtained from surgical specimens were immediately frozen in liquid nitrogen until RNA isolation. Total RNA was extracted from 14 tumor samples with low SUV and 24 tumors samples with high SUV by using ISOGEN (Nippon Gene, Tokyo, Japan). The amount and quality of RNA were assessed using a UV spectrophotometer. cDNA microarray analysis was performed using IntelliGene^® ^Human Cancer CHIP Ver. 4.0 (Takara, Kusatsu-shi, Japan). Briefly, 4 μg of total RNA was used for double-stranded cDNA probe synthesis with a T7 oligo (dT) primer. Each cDNA fragment was then subjected to RT amplification that incorporated aminoally-UTP (Ambion), which was coupled with either Cy3 or Cy5 (Amersham Biosciences), and purified through a microcon 30 spin colum (Millipore). The generated RNA was used as a probe for hybridization in IntelliGene^® ^Human Cancer CHIP Ver. 4.0, as previously described[14]. The cDNA chip arrayed and immobilized with approximately 890 cDNA fragments of human genes, which are composed of approximately 590 kinds of identified human oncogenes and approximately 300 kinds of the cDNA fragments prescreened by the differential display method between cancer tissue and corresponding normal tissue. For each gene identified by the differential display method, a cDNA region approximately 300 bp in length, which has minimal homology with other genes registered in the cDNA database, was spotted on the DNA microarray chip [15].

We defined genes with Cy3:Cy5 ratios of 3.0 or greater in signal intensity as up-regulated genes because of the following reasons:

We first identified 20 genes, that showed the Cy3:Cy5 ratios of 1.7 or greater, as the candidate genes that were upregulated in the high SUV tumors (Table 2). In the two genes that showed the Cy3:Cy5 ratios of 3.0 or greater, i.e., CENP-F and CDC6, we could validate their upregulation in the high SUV tumors by RT-PCR as mentioned below.

**Table 2:** Twenty genes that were identified as upregulated in tumors of the high SUV group compared with those of the low SUV group

Rank	Symbol	Gene name	Genbank Accession No.	High/Low ratio^a)^	Function
1	CENPF	centromere protein F (mitosin)	NM016343	3.66	cell-cycle regulation
2	CDC6	cell division cycle 6	NM001254	3.36	cell-cycle regulation
3	gtf2b	general transcription factor IIB	M76766	2.79	transcription initiation
4	Fgr	Gardner-Rasheed feline sarcoma viral oncogene homolog	NM005248	2.49	mast cell activation
5	GAPDHS	glyceraldehyde-3-phosphate_dehydrogenase	NM002046	2.18	carbohydrate metabolism
6	Oaz1	ornithine decarboxylase 1	NM002539	2.14	regulation of polyamine synthesis
7	ACTA1	actin, alpha 2, smooth muscle, aorta	NM001613	2.1	cell motility, structure
8	KRT5	keratin 5	NM000424	2.05	type 2 cytokeratine
9	RCC1	chromosome condensation 1	NM001269	2.03	cell-cycle regulation
10	BAK1	BCL2-antagonist/killer 1	NM001188	1.96	anti- or pro-apoptotic regulators
11	MMP9	matrix metalloproteinase 9	NM004994	1.95	embryonic development and tissue remodeling
12	MAP2K5	mitogen-activated protein kinase kinase 5	NM002757	1.94	Kinase related to signal cascade
13	MAP2K2	mitogen-activated protein kinase kinase 2	NM030662	1.93	Kinase related to signal cascade
14	Litaf	LPS-induced TNF-alpha factor	NM004862	1.9	proinflammatory cytokine
15	COL7A1	collagen, type VII, alpha 1	NM000094	1.85	epithelial adhesion complex
16	HRMT1L2	HMT1 (hnRNP methyltransferase, S. cerevisiae)-like 2	NM001536	1.84	catalyzes protein methyl-transferase modification
17	CXCL13	small inducible cytokine B subfamily, member 13	NM006419	1.82	migration of B lymphocytes
18	PLAU	plasminogen activator, urokinase	NM002658	1.78	serine protease
19	CDC37	cell division cycle 37	NM007065	1.77	cell-cycle regulation
20	Pax	paxillin	NM002859	1.74	cell adhesion of the extracellular matrix

a) High/low ratio; the ratio of the quantitative value of the gene expression in tumors of the high SUV group to that in tumors of the low SUV group.

On the other hand, in other candidate genes, e.g., gtf2b (Cy3:Cy5 ratio 2.79), KRT5 (Cy3:Cy5 ratio 2.05), MMP9 (Cy3:Cy5 ratio 1.95), and PLAU (Cy3:Cy5 ratio 1.78), their up-regulation could not be validated by means of RT-PCR. Furthermore, a housekeeping gene GAPDH was also ranked in the candidate genes with a Cy3:Cy5 ratio of 2.18 by cDNA microarray analysis. Therefore, we chose the cut-off value of 3.0.

### Validation of microarray data with RT-PCR

Two candidate genes (CENP-F and CDC6) detected on the cDNA microarray screening were selected for the validation of the microarray results. Total RNA was extracted from 100 μg of frozen tumor tissue obtained from each primary breast cancer specimens. Reverse transcription for cDNA was performed from 5 μg of total RNA. PCR was performed using dNTP, *Taq *polymerase primer pair, and cDNA using a thermal cycler. The primer sequences of CENP-F were 5'-CGAAGAACAACCATGGCAACTCG-3' and 5'-TTCTCGGAGGATGGTGCCTGAAT-3'. The primer sequences of CDC6 were 5'-GCGATGACAACCTATGCAACAC-3' and 5'-TTGGTGGAGAACAAGGAGGTAAA-3'.

The expression value for each gene was normalized against that of β-actin. Results are presented as the mean ± standard error (SE). Statistical analysis was performed using the Student's *t *test.

### Case selection for a validation study

To validate the expression levels of candidate genes at the protein level, we performed an immunohistochemical study. A cohort of 47 patients was selected from those who received surgical therapy to node-negative primary breast cancer from August through December in 2006. The primary cancers exhibited low SUV (mean 2.2 ± 1.0 SD) in 20 patients and high SUV (mean 7.8 ± 3.4 SD) in 27. The patients' ages (mean 53.2 ± 10.0 SD and mean 55.6 ± 10.1 SD, respectively), tumor size (mean 2.4 ± 2.9 SD and mean 3.1 ± 2.1 SD, respectively), ER (positive in 85% and 59%, respectively), and HER2 status (positive in 10% and 26%, respectively) did not differ significantly between the low- and high-SUV groups (Table 3). Molecular subtypes were also evaluated as described below.

**Table 3:** Comparision of clinicopathological features of patients and immunohistochemical results of biomarkers between the low SUV group and the high SUV group in 47 cases of node-negative breast cancer

Parameters		High SUV group (n = 27)	Low SUV group (n = 20)	p-value
SUV	Ave± SD	7.8± 3.4	2.2± 1.0	<0.0001
Age	Ave± SD	55.6± 10.1	53.2± 10.0	0.42
Tumor size	Ave± SD	3.1± 2.1	2.4± 2.9	0.33
Nuclear grade	1 and 2	8	15	0.004
	3	19	5	
ER	<10%	11	3	0.06
	10% ≦	16	17	
HER2	0,1+,2+ (FISH-)	20	18	0.17
	3+ or FISH+	7	2	
CENP-F	Positive	20	9	0.04
	Negative	7	11	
n-CDC6	Positive	13	10	0.9
	Negative	10	10	
c-CDC6	Positive	9	7	0.9
	Negative	18	13	
Ki67	Positive	25	12	0.02
	Negative	2	8	
p53	Positive	9	2	0.06
	Negative	18	18	
Molecular subtypes	Luminal A	16	17	0.05
	HER2	7	2	
	Triple negative	4	1	

Abbreviations; SUV, Standardized uptake value; ER, Estrogen receptor; HER2, c-erbB2; FISH, Fluorescence in situ hybridization; SD, standard deviation; CENP-F, Centromere protein-F; SUV, Standardized uptake value; n-CDC6, nuclear staining of Cell devision cycle 6; c-CDC6, cytoplasmic staining of Cell devision cycle 6

### Tissue microarray (TMA) construction

Hematoxylin and eosin (HE)-stained sections of archival pathological primary breast cancer specimens were reviewed. These specimens were resected from patients between January 1990 and December 1995. The histological diagnosis including histological type and nuclear grade was confirmed for all cases. For the 253 available cases of primary breast cancer, two tissue cores with a diameter of 2.0 mm were punched out from representative areas in 253 archival pathological tissue blocks. TMA was constructed by the method described previously [16]. One TMA block contained a maximum of 40 tissue cores, and 8 TMA sets were prepared for the present study. We then cut 4-μm-thick sections from the blocks.

### Immunohistochemistry

We cut 4-μm thick sections from routinely processed, formalin-fixed paraffin-embedded tissue blocks or TMA blocks and mounted them on silane-coated slides. Immunohistochemistry with anti-human antibodies against centromere protein F (CENP-F) (ab90, 1/200 dilution; Abcam, Cambridge, MA), phosphorylated-CDC6-S54 (CDC6) (AP3058a, 1/200 dilution; Abgent, San Diego, CA), Ki67 (clone: MIB1, ready-to-use, Dako, Glostrup, Denmark), and p53 (clone: DO-7, 1/200 dilution, Dako) was performed by the streptavidin-biotin-peroxidase complex method or the EnVision (Dako) method. To assess CENP-F and CDC6 expressions, normal skin tissues were used as the positive control. ER and HER2 were also immunohistochemically analyzed using specific primary antibodies (Dako) as described previously [16,17].

Staining results were independently assessed by 2 of 3 observers (S.U, S.Y, and H.T). When there was a difference in judgment, consensus was reached over a discussion microscope. Immunohistochemical results were assessed based on both the intensity and proportion of cancer cells with nuclear, cytoplasmic, and/or membrane staining. The intensity of nuclear staining were 0 (null), 1+ (faint), and 2+ (strong). Positivity was defined as nuclear staining of 10% or more cancer cells with a strong intensity for CENP-F, Ki67, p53, ER, and PgR. For CDC6, expression patterns in cancer cells were classified into nuclear and cytoplasmic. The detection of nuclear and cytoplasmic stainings in 10% or more cancer cells was defined as positivity of nuclear CDC6 (nCDC6) and cytoplasmic CDC6 (cCDC6), respectively. For HER2, a score of 3+ detected by Herceptest, or gene amplification detected by means of fluorescence *in situ *hybridization were defined as positive [16]. According to the status of ER and HER2, the 47 cases were classified into molecular subtypes, i.e., ER+/HER2- (luminal A subtype), ER+/HER2+ (luminal B subtype), ER-/HER2+ (HER2 subtype), and ER-/HER2- (triple-negative subtype).

### Statistical analysis

Statistical analysis was performed using StatView 6.0 software. Positivity of molecular expression was compared between the groups. Cox's proportional hazard regression models were used to assess the prognostic contribution of clinical variables and biomarkers. Hazard ratios were verified by all-possible-subset analyses. A *p *value of less than 0.05 was considered significant.

## Results

### Identification of genes correlated by tumoral SUV

**Figure 1:**
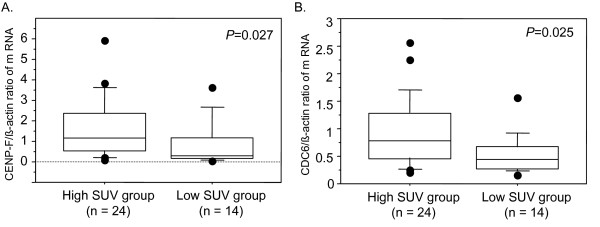
**The relative expression levels were calculated against the levels of β-actin expression**. Mann-Whitney test was performed to confirm the statistical significance of expression levels of CENP-F and CDC6 in breast cancer tissues between patients in low SUV group and those in high SUV group. The bars represent (A) the ratio of the expression level of CENP-F to that of β-actin expression level and (B) the ratio of the expression level of CDC6 to that of β-actin. Empty and filled bars represent a case of the low SUV group and high SUV group, respectively.

We calculated the ratio of the quantitative value of gene expression in the high SUV group to that in the low SUV group for each gene by cDNA microarray analysis. We identified 20 genes, the expression levels of which were 1.7-fold or higher in the high SUV group than in the low SUV group (Table 2). Of these, the expression levels of the CENP-F and CDC6 were threefold or higher in the high SUV group than in the low-SUV group (Table 2). Quantitative RT-PCR analysis using total RNA identical to that used for cDNA microarray analysis was performed. Results revealed that the average ratios of CENP-F and CDC6 expressions relative to that of β-actin expression were significantly higher in the high SUV group (1.6 ± 0.3 SE and 0.9 ± 0.1 SE) than in the low SUV group (0.8 ± 0.3 SE and 0.5 ± 0.1 SE) (*p *= 0.027 and 0.025, respectively) (Figure 1).

### Validation of gene expression data by immunohistochemistry

We assessed a cohort of 47 breast cancers for validation by immunohistochemistry. Of these, 20 cancers with low SUV comprised 15 tumors of nuclear grade 1/2 and 5 of nuclear grade 3, while 27 cancers with high SUV included 8 tumors of nuclear grade 1/2 and 19 of nuclear grade 3 (Table 2). Immunohistochemical results of 20 low SUV and 27 high SUV cancers for CENP-F, CDC6, Ki67, p53 and molecular subtypes are also shown in Table 3.

**Figure 2:**
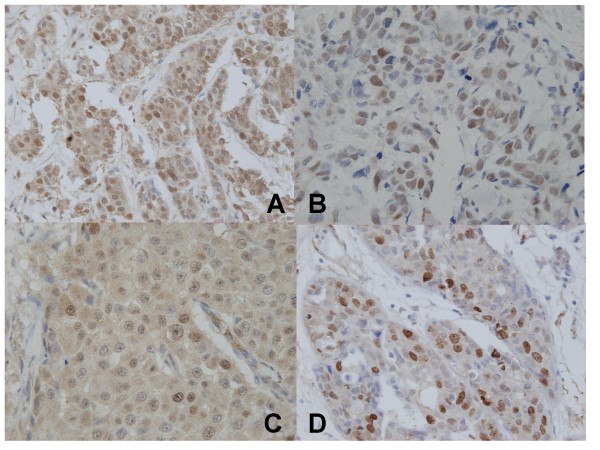
**CENP-F (A) and Ki67 (D) exhibited immunohistochemically positive staining of nucleus in invasive ductal cancer of the breast**. CDC6 revealed 2 patterns of nuclear and cytoplasmic expressions in invasive ductal carcinoma of the breast. Positive nuclear and cytoplasmic stainings of CDC6 are presented in (B) and (C), respectively.

CENP-F is a nuclear protein regulating cell cycle that is overexpressed in the M phase. The positivity of the CENP-F expression in the high SUV group (74%, 20 of 27) was significantly higher than that in the low SUV group (45%, 9 of 20) *(p *= 0.04) (Figure 2).

CDC6 expression is reportedly present in nuclei throughout the G1 phase of the cell cycle. CDC6 translocates to the cytoplasm after being activated by phosphorylation and is mainly present in the S phase of the cell cycle. Nuclear and cytoplasmic CDC6 expressions were positive in 23 (49%) and 16 (34%) of the 47 breast cancers and were detected simultaneously in 11 cases (23%). However, these expressions did not significantly differ between the high and low SUV groups of primary breast cancers (*p *= 0.9 in both cases) (Figure 2).

Strong nuclear staining of Ki67 and p53 in >10% of the cancer cells was detected in 37 (79%) and 11 (23%) of the breast cancers, respectively. Both were significantly or almost significantly more frequent in the high SUV group than in the low SUV group (*p *= 0.02 and 0.06, respectively).

Seven (78%) of 9 tumors of the HER2 subtype, 4 (80%) of 5 tumors of the triple-negative subtype were categorized in the high SUV group, while 16 (48%) and 17 (52%) of 33 tumors of the luminal-A subtype were divided in the high and low SUV groups, respectively. Tumors of the HER2 or triple-negative subtypes were more frequently included in the high SUV group than the luminal-A subtype (*p *= 0.05) (Table 3).

### Associations of clinicopathological factors and biomarkers with clinical outcome

Tumor invasion size, histological type, nuclear grade, nodal status, ER, HER2, CENP-F, Ki67, and p53 were evaluated in the 253 primary breast cancers subjected to the TMA analyses. There were 81 (32%) tumors with invasive size of 2 cm or less and 172 (68%) tumors with invasive size of more than 2 cm, 232 (92%) tumors with invasive ductal carcinoma and 21 (8%) tumors with other types, 88 (35%) tumors with nuclear grade 3 and 154 (61%) tumors with nuclear grade 1/2, and 112 (44%) tumors with nodal metastasis and 141 (56%) node-negative tumors. The positivity of ER and HER2 were 58% (148 of 253) and 10% (26 of 253), respectively.

Clinical follow-up data were retrieved in 253 patients in disease-free survival (DFS) and 251 patients in overall survival (OS) with a mean follow-up period of 76 months (± 39.3 SD) at the time of the analysis. Because CENP-F, Ki67, and p53 were correlated with or nearly correlated with the SUV status of the primary breast cancer, we examined the frequency of their positivity and their prognostic implication for these cases. The positivity of CENP-F, Ki67, and p53 were 49% (124 of 253), 34% (87 of 253), and 18.6% (47 of 253), respectively. Univariate analyses revealed that a tumor size of 2 cm or more (HR = 6.7, *p *< 0.0001), nodal positivity (HR = 4.79, *p *< 0.0001), nuclear grade 3 (HR = 3.96, *p *< 0.0001), a high HER2 level (HR = 2.11, *p *= 0.031), positive Ki67 (HR = 2.25, *p *= 0.0016), positive p53 (HR = 2.35, *p *= 0.0024), and positive CENP-F (HR = 3.92,*p *< 0.0001) revealed higher recurrence rates. Furthermore, in a multivariate analysis, not only tumor size of 2 cm or more (HR = 4.49, *p *= 0.0047) and nodal positivity (HR = 4.1, *p *< 0.0001) but also positive CENP-F (HR = 2.94, *p *= 0.0015) and Ki67 (HR = 2.05, *p *= 0.011) showed an independent impact on the DFS of patients (Table 4).

**Table 4:** Univariate and multivariate analyses of prognosis using pathological factors and biomarkers of primary breast cancer

	Disease-free survival					
	
Variable	Univariate			Multivariate		
		
	HR	95% CI	*p*-value	HR	95% CI	*p*-value
Tumor size (≧2 cm vs <2 cm)	6.7	2.68–16.72	<0.0001	4.49	1.56–12.74	0.0047
Nodal status (+ vs -)	4.79	2.73–8.38	<0.0001	4.1	2.18–7.74	<0.0001
Nuclear grade (Grade 3 vs 1–2)	3.96	2.32–6.76	<0.0001	1.69	0.92–3.13	0.094
CENP-F (positive vs negative)	3.92	2.19–7.03	<0.0001	2.94	1.51–5.72	0.0015
p53 (positive vs negative)	2.35	1.36–4.09	0.0024	1.38	0.75–2.52	0.3
Ki67 (10% ≦ vs 10% >)	2.25	1.36–3.72	0.0016	2.05	1.18–3.56	0.011
HER2 (3+,FISH+ vs 0,1+,2+(FISH-))	2.11	1.07–4.16	0.031	1.79	0.84–3.8	0.13
ER (positive vs negative)	1.54	0.94–2.52	0.087		n/a	

	Overall survival					
	
	Univariate			Multivariate		
		
Variable	HR	95% CI	*p*-value	HR	95% CI	*p*-value

Nodal status (+ vs -)	9.83	2.58–15.71	0.0041	11.88	1.45–97.09	0.021
Nuclear grade (Grade 3 vs 1–2)	3.73	1.25–11.15	0.0019	2.51	0.72–8.04	0.15
CENP-F (positive vs negative)	3.35	1.05–10.72	0.042	2.05	0.52–8.09	0.31
Tumor size (≧2 cm vs <2 cm)	2.28	1.3–3.99	0.0039	1.37	0.72–2.64	0.34
HER2 (3+,FISH+ vs 0,1+,2+(FISH-))	3.02	0.84–10.85	0.09		n/a	
Ki67 (10% ≦ vs 10% >)	2.22	0.78–6.36	0.13		n/a	
p53 (positive vs negative)	1.71	0.48–6.13	0.41		n/a	
ER (positive vs negative)	1.26	0.44–3.62	0.67		n/a	

Abbreviations; HR, hazard ratio; CI, confidence interval; vs, versus; n/a, not applicable; positive, strong staining (2+) in 10% of cancer cells;negative, others

With regard to OS, univariate analyses revealed that nodal positivity (HR = 9.83, *p *= 0.0041), nuclear grade 3 (HR = 3.73, *p *= 0.0019), tumor size of 2 cm or more (HR = 2.28, *p *= 0.0039), and positive CENP-F (HR = 3.35, *p *= 0.042) exhibited a significantly higher rate of cancer death. A multivariate analysis with regard to OS revealed that nodal positivity (HR = 11.88, *p *= 0.021) only remained an independent predictor of patient outcome (Table 4).

**Figure 3:**
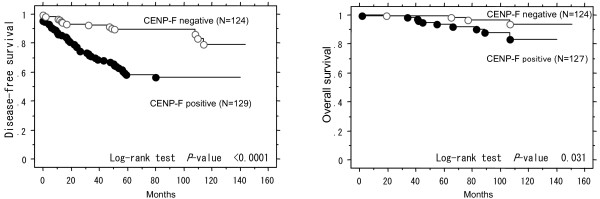
**Kaplan-Meier curves of disease-free survival in 253 patients and overall survival in 251 patients with primary breast cancer as having low or high rates of CENP-F positive cancer cells**. A cut-off point of 10% positive nuclei was used. The *P *value was calculated using the log-rank test.

In the Kaplan-Meier curves of DFS and OS in the 253 patients having primary breast cancer, curves for the CENP-F-positive subgroup were significantly different from those for the CENP-F-negative subgroup (*p *< 0.0001 and *p *= 0.031, respectively) (Figure 3).

## Discussion

We compared the gene expression profiles between tumors having a high level of SUV and those having a low level of SUV, and we identified 20 candidate genes in high SUV tumors with an mRNA expression level of greater than 1.7-fold that in low SUV tumors by cDNA microarray analysis. Candidate genes included genes related to cell-cycle function, transcriptional initiation, carbohydrate metabolism, and metastatic potential (Table 2). We confirmed that the 2 candidate genes CENP-F and CDC6 were upregulated in the high SUV tumors by means of quantitative RT-PCR analysis in the study of 47 resected tumors.

SUV levels are reported to be correlated with multiple factors including glucose metabolism, mitotic activity index, and tumor cell density [13,18-20].

In the previous studies, we demonstrated that tumors with high SUV comprised larger tumor size of invasion and nuclear grade 3 in comparison with those with low SUV, and for primary tumors with invasive size of 2 cm or less, nuclear grade was the independent factor that influenced SUV [9].

In the present study, we used cDNA microarray analysis to demonstrate the strong relationship between SUV and expression of CENP-F and CDC6 as cell cycle-associated genes.

CENP-F is a nuclear protein associated with the centromere-kinetochore complex, which monitors chromosome-microtubule interactions and serves as the source of checkpoint signals. CENP-F gradually accumulates during the cell cycle until it attains peak levels at the G2/M phase and rapidly degrades upon the completion of mitosis in vitro [21]. Thus, its expression level is considered to be involved in M phase progression, which gradually increases throughout the S, G2, and M phases of the cell cycle but is absent in G0 and G1 [21,22].

CDC6 is a regulator of the early initiation of DNA replication of cancer cells in the S phase. CDC6 licenses DNA replication, and once it is bound to the origin recognition complex at the origins of replication, it commences recruitment, together with Cdt1 and the MCM complex[23,24]. Transcription of this protein was reported to be regulated in response to mitogenic signals through a transcriptional control mechanism involving E2F proteins. The phosphorylated CDC6 is considered to be localized in cell nuclei during the G1 phase, but it translocates to the cytoplasm at the start of the S phase [25,26].

To confirm the expression of CENP-F and CDC6 immunohistochemically, we conducted a cohort study between the low and high SUV groups of node-negative breast cancer. We also examined the expression of Ki67 and p53 as biomarkers of tumor proliferation and aggressiveness of breast cancer.

Ki67 and p53 are well-known biomarkers of tumor proliferation and aggressiveness[2]. Ki67 is a nuclear antigen of unknown function and is detectable in cancer cells entering the cell cycle but not in the G0 phase[27]. p53 is also implicated in the checkpoint of cell-cycle progression in combination with DNA repair and/or induction of apoptosis. Most of the mutant p53 proteins are accumulated in the nuclei and can be immunohistochemically detected[27].

We could demonstrate significant correlations between high SUV tumors and high CENP-F levels; however, no correlations could be observed between high SUV tumors with both nuclear and cytoplasmic CDC6 expressions.

According to the CENP-F expression level, some investigators have reported that CENP-F is immunohistochemically correlated with highly proliferative cancer cells and poorer prognosis[21,22,28]. Clark et al. reported that high CENP-F expression and tumor size were independent predictors of overall survival in a multivariate analysis containing other prognostic factors such as tumor size, patients age, hormonal receptors and S-phase fraction, in 386 node-negative breast cancer patients [28].

In a validation study of the DNA microarray dataset using a 70-gene prognosis classifier (Mammaprint^®^), O'Brien et al. reported that immunohistochemical expression of CENP-F was an important predictor among the genes highly expressed in breast tumors of patients with poor overall survival[22]. The results in the present study were mostly compatible with the previous studies discussed above[22,28].

In the follow-up data using TMAs of primary breast cancer, CENP-F expression was correlated with a higher recurrence rate and had a tendency of correlation with mortality by cancer. As shown in Table 3, high levels of CENP-F retained prognostic significance together with tumor size and nodal metastasis in a multivariate analysis.

The mRNA level of CDC6 was significantly higher in the high SUV group than the low SUV group of primary breast cancer (*p *= 0.025, Figure 1). However, the immunohistochemical expression of CDC6 did not significantly differ between both the groups. Anja et al. reported that the increase in the CDC6 mRNA observed in the absence of p53 was required for the enhanced proliferation of MCF-7 cells[24]. CDC6 might be a part of a p53 protein network regulating the cell cycle of cancer cells. Further, there have been a few reports that supported the clinical impact of immunohistochemical expression of CDC6[29,30]. Nevertheless, the clinical implications of the immunohistochemical expression of CDC6 are still undetermined.

High Ki67 levels (> 10% of the labeling index) were detected more frequently in the high SUV group than in the low SUV group of primary breast cancer (*p *= 0.02, Table 4) and correlated with shorter disease-free survival in the univariate and multivariate analyses of DFS (*p *= 0.0016 and 0.011, respectively, Table 4).

High p53 levels marginally correlated with primary breast cancers with high SUVs (*p *= 0.06, Table 4); however, they were significantly related with poorer prognosis in the univariate analysis of DFS (*p *= 0.024, Table 4). We assume that primary breast cancer with high SUV is a characteristic of aggressiveness originated from the p53 alteration, but it seems unlikely that the immunohistochemical measurement of p53 could sufficiently provide clinically useful results because it does not exhibit p53 nonsense point mutations or deletions in p53[31,32].

Interestingly, the majority of tumors with HER2 and triple-negative subtypes were included in the high SUV group, while tumors of the luminal-A subtype displayed both the high SUV and low SUV groups (Table 3). We suggested tumors of the HER2 and triple-negative subtypes might feature high proliferation activity, while the luminal-A subtype could be comprised of tumors with various proliferation activity. We need further long-term follow-up survey of patients and compare the prognostic impact of SUV between the high and low SUV groups of the luminal-A tumors.

## Conclusion

**Figure 4:**
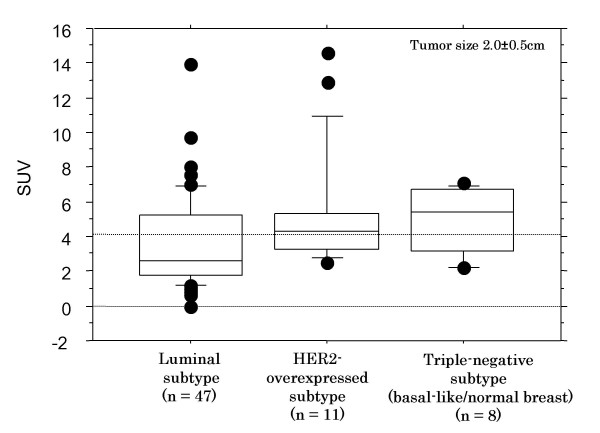
**This figure shows the relationship of SUV with breast cancer subtypes including ER+/HER2- (luminal-A) tumors, HER2-overexpressing tumors (HER2 subtype), and ER-/PR-/HER2- (triple-negative, including basal-like and normal breast types) tumors**. Only tumors with diameter between 1.5 cm and 2.5 cm were examined. HER2- tumors and triple-negative tumors exhibit higher SUV levels of 5.0 (5.3 SD) and 5.0 (1.9 SD), respectively, compared to luminal-A tumors with SUV of 3.6 (2.7 SD) (*p *= 0.05 and 0.002, respectively).

The present data demonstrated that high SUV in early-stage breast cancer represents a specific biological status of tumor cell proliferation with the upregulation of particular genes. High expression levels of the CENP-F appeared to be the molecular background of higher proliferative activity, and they were correlated with high SUV in breast cancer. Therefore, high SUV levels in primary breast cancer may serve as a predictor of proliferation activity and aggressiveness of tumor cells (Figure 4).

We might be able to pretherapeutically elucidate the biological characteristics of primary breast cancers by means of FDG-PET/CT scan, and monitoring the SUV in primary breast cancer would be informative for treatment choice in the PST setting. Furthermore, FDG PET imaging may be helpful to predict or identify the patients who will show a pathological complete response following neoadjuvant therapy.

## Abbreviations

(CENP-F): centromere protein F; (SUV): standardized uptake value; (FDG PET/CT): 18F-fluorodeoxyglucose positron emissiontomography/computed tomography; (FDG): ^18^F-fluorodeoxyglucose; (PST): primary systemic therapy; (ROI): region of interest; (TMA): Tissue microarray; (nCDC6): nuclear CDC6; and (cCDC6): cytoplasmic CDC6.

## Competing interests

The authors declare that they have no competing interests.

## Authors' contributions

SU performed the planning, acquisition of data, analysis of data, and writing of the manuscript. HT and SY (pathologists) performed the planning, interpretation of data, and the manuscript in co-operation with SU. JY, KF, and SU (breast surgeons) performed surgery and the statistic analysis. KT, JI, and YA (radiologists) performed the evaluation of tumoral SUV levels and data acquisition. NK (biochemist) performed the statistic analysis. TK (oncologist) constructed the TMA and performed data analysis. HM participated in its design and coordination in co-operation with SU and HT. All authors read and approved the final manuscript.

## Pre-publication history

The pre-publication history for this paper can be accessed here:


